# Cardiovascular risk factors and the impact on prognosis in patients with chronic kidney disease secondary to autosomal dominant polycystic kidney disease

**DOI:** 10.1186/s12882-021-02313-1

**Published:** 2021-03-25

**Authors:** José Luis Gorriz, David Arroyo, Luis D’Marco, Roser Torra, Patricia Tomás, María Jesús Puchades, Nayara Panizo, Jonay Pantoja, Marco Montomoli, José Luis Llisterri, Vicente Pallares-Carratalá, José Manuel Valdivielso

**Affiliations:** 1 Department of Nephrology, University Clinic Hospital, INCLIVA, University of Valencia, Av Blasco Ibañez 17, 46010 Valencia, Spain; 2 Department of Nephrology, Hospital General Universitario Gregorio Marañón, Madrid, Spain; 3 Inherited Kidney Diseases, Nephrology Department, Fundació Puigvert, Instituto de Investigaciones Biomédicas Sant Pau (IIB-Sant Pau), Medicine Department-Universitat Autónoma de Barcelona, REDinREN, nstituto de Investigación Carlos III, Barcelona, Spain; 4 Department of Nephrology, University Dr Peset Hospital, Valencia, Spain; 5 Clinica Vallada, Calle San Ramón 2, 46691 Valencia, Spain; 6 Health Surveillance Unit, Castellon Mutual Insurance Union, Castellon, Spain. Department of Medicine, Jaume I University, Castellon, Spain; 7 Vascular and Renal Translational Research Group, UDETMA, REDinREN del ISCIII, IRBLleida, Lleida, Spain, 2 Statistics Department, University of Lleida, Lleida, Spain

**Keywords:** Autosomal dominant polycystic kidney disease, Chronic kidney disease, Cardiovascular disease, Nephropathy

## Abstract

**Background:**

Autosomal dominant polycystic kidney disease (ADPKD) is the most frequent hereditary renal disease. There is an increased rate of cardiovascular disease (CVD) in ADPKD. In this study, we evaluate the prevalence of cardiovascular risk factors, the achievement rates for treatment goals and cardiovascular events (CVE) in ADPKD and their relations with asymptomatic CVD in CKD from other etiologies (CKDoe) and controls.

**Methods:**

We evaluated 2445 CKD patients (2010–2012). The information collected was: clinical, anthropometric and analytical parameters, treatments and CVD evaluation (intima-media thickness (IMT), atheromatous plaque presence and ankle-brachial index (ABI)). Laboratory, vital status, CVE and hospitalizations were collected for 4 years.

**Results:**

ADPKD patients had a worse renal function and worst achievement of blood pressure, higher parathormone levels but lower proteinuria compared to CKDoe. ADPKD patients presented lower IMT values than other groups, however, an intermediate rate of pathologic ABI and atheromatous plaque was present. More than half of the patients received statins, achieving LDL-c levels < 100 only in 50 and 39.8% of them (ADPKD and CKDoe respectively). The number of CVE during the follow-up period was low. In adjusted Cox regression model, ADPDK had the lowest occurrence of CVE of all three groups (HR:0.422, 95%CI 0.221–0.808, *p* = 0.009).

**Conclusion:**

ADPKD patients show intermediate control rates of CVD. A better control of CVD risk seems to be related with a lower load of CVD compared to other groups, which may lead in the long term to a better prognosis. Further investigation is necessary to determine cardiovascular prognosis in ADPKD.

**Supplementary Information:**

The online version contains supplementary material available at 10.1186/s12882-021-02313-1.

## Introduction

Cardiovascular disease (CVD) is the leading cause of death in patients with chronic kidney disease (CKD) from earlier stages of the disease compared to the general population [1, 2]. Consequently, CKD has been considered an important cardiovascular risk factor in several international guidelines. In this regard, several factors have been postulated to contribute to this increased risk, mainly, age at CKD initiation and comorbidities such as arterial hypertension and diabetes, followed by other related conditions such as obesity or dyslipidemia [3]. On the other hand, CKD per se produces several alterations that have a direct impact on vascular health, such as proteinuria, anemia, metabolic acidosis or mineral bone disease (MBD). These lastly is known as non-traditional or uremic related factors. Some of these factors may partially explain the higher rates of both asymptomatic CVD and cardiovascular events (CVE) among CKD patients.

**Fig. 1:**
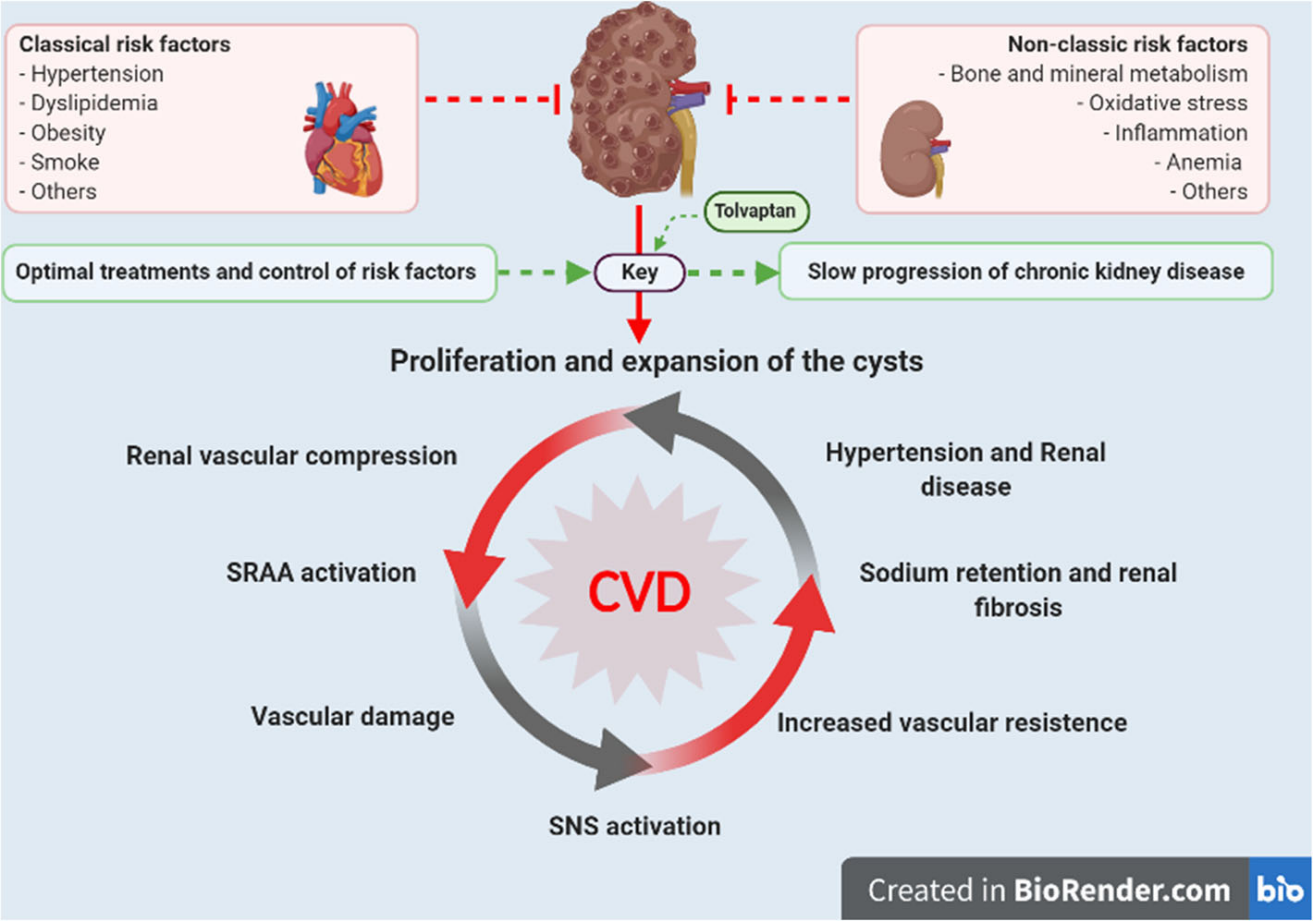
Pathophysiological mechanisms associated with the initiation and progression of chronic kidney disease in autosomal dominant polycystic kidney disease. This schematic figure shows the negative influence of classical and renal or uremic-related cardiovascular risk factors in patients affected by ADPKD. The key to management these patients depend of the optimal approach and good control of these associated risk factors. The uses of novel therapies as vasopressin V2-receptor antagonist (Tolvaptan) may delay the progression of the renal disease specifically at the first step of the pathophysiological mechanisms related to proliferation and expansion of the cyst. Finally, if these measures are not taking in the early stages of ADPKD, a cascade of events can develop serious consequences. In addition, an activation of SRAA and SNS may produce vascular damage, increase the vascular resistance and renal fibrosis. All these activated mechanisms induce a chronic and irreversible damage in the kidneys. Abbreviations: SRAA, renin – angiotensin – aldosterone system; SNS, sympathetic nervous system; ADPKD, autosomal dominant polycystic kidney disease; CVD, cardiovascular disease; CKD, chronic kidney disease

Autosomal dominant polycystic kidney disease (ADPKD) is the most frequent hereditary renal disease and accounts for approximately 6 to 10% of the patients initiating renal replacement therapies (RRT) worldwide [2, 4]. Although ADPKD is not usually associated with diabetic or hypertensive nephropathy, there is an increased in the rate of CVD in ADPKD patients being the first cause of death (Fig. 1) [5, 6]. It should be noted that there is evidence that asymptomatic CVD predicts the incidence of CVE [7], but data on evaluation and management of cardiovascular risk in ADPKD are limited.

This study evaluates the prevalence of classic cardiovascular risk factors in ADPKD, its relationships to asymptomatic CVD and treatment trends in ADPKD patients in a large cohort of CKD patients and controls without previous CVD.

## Material and methods

### Study design and participants

This study is a sub-analysis of the NEFRONA project, which consists of a large prospective multicenter cohort designed to evaluate asymptomatic CVD in patients at different stages of CKD without previous CVE. Extensive information on methodology and initial results has been published previously [8, 9]. In summary, from October 2010 to June 2012, 2445 patients with CKD (937 CKD stage 3, 820 CKD stages 4–5 and 688 in dialysis) were enrolled in 81 hospitals and dialysis units throughout Spain, along with 559 patients without CKD used as controls, also without previous CVE selected from 14 Spanish primary care centers. Controls did not have CKD but were not completely healthy subjects, as they attended primary care due to different comorbidities.

A biannual follow-up was conducted over 4 years. The patients signed the informed consent and the study protocol was approved by the Ethics Committee of the Arnau de Vilanova University Hospital.

**Fig. 2:**
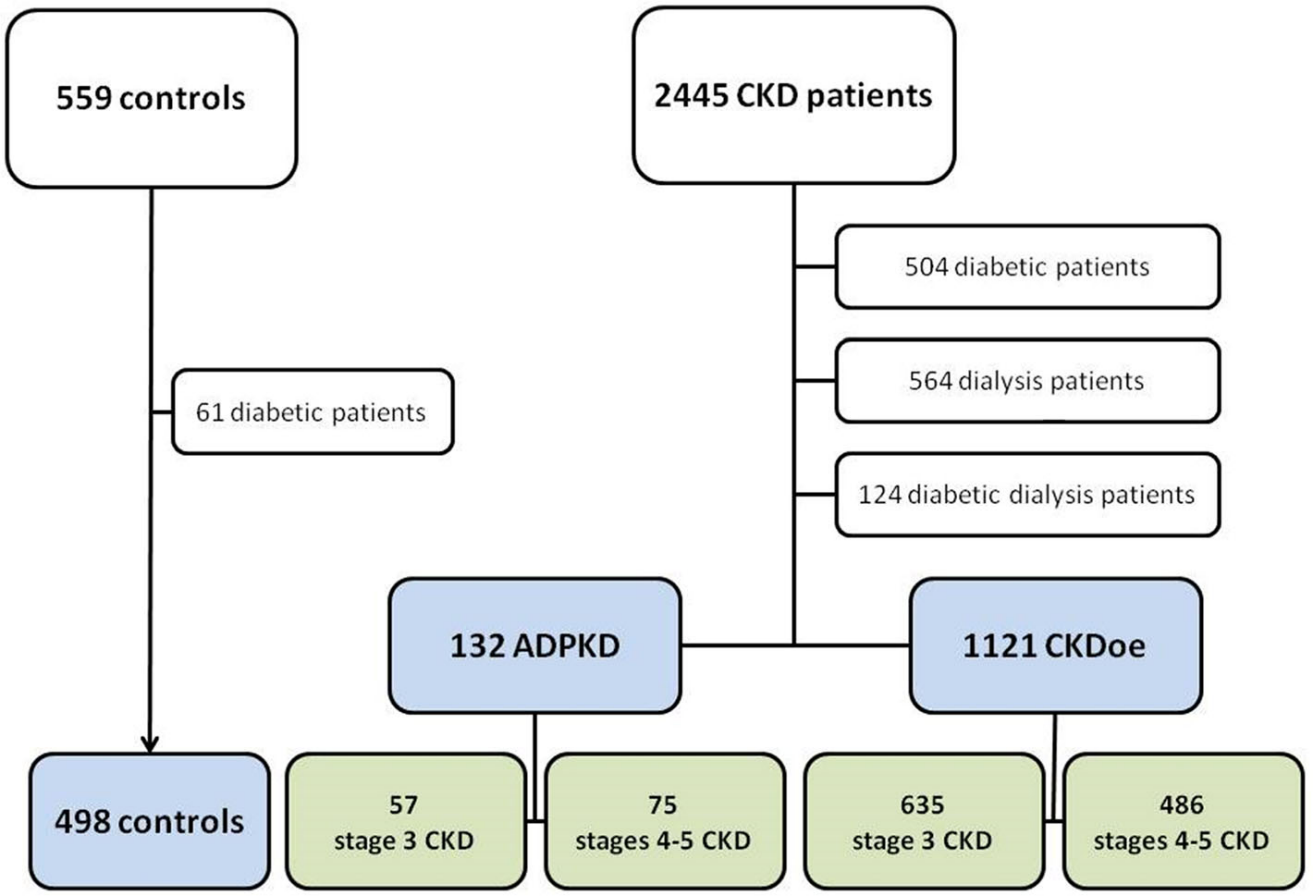
Flowchart describing the patient selection. Abbreviations: ADPKD, autosomal dominant polycystic kidney disease; CKD, chronic kidney disease; CKDoe, CKD of etiologies other than ADPKD

For this sub-study, 1192 patients with diabetes or on dialysis were excluded to avoid the confusing effect of these circumstances. Finally, a total of 1.751 patients were included: 132 patients with ADPKD were compared with 1121 patients with CKD of other etiologies (CKDoe) and 498 controls (Fig. 2).

### Clinical data

The information collected at the baseline and at 24 months was as follow: clinical and anthropometric data, analytical parameters (including renal function, metabolic profiles, anemia, CKD-related MBD and inflammation parameters), pharmacological treatments and the evaluation CVD using the intima-media thickness (IMT), the presence of carotid atheromatous plaque and the ankle-brachial index (ABI). All anthropometric and cardiovascular evaluations were performed using a standardized method by three experienced itinerant teams. The occurrence of fatal and non-fatal CVE was evaluated biannually, as well as the death from other causes and kidney transplantation. Laboratory data, vital status, cardiovascular events and hospitalizations were collected every 2 years and at the end of the study (48 months).

Patients with ADPKD were compared to non-CKD patients and patients with CKDoe. In order to assess the specific impact of ADPKD and exclude the added effect of advanced CKD, a secondary analysis was performed comparing only patients with CKD stage 3 an ADPKD with controls.

The adequacy of cardiovascular risk factors control across groups was compared (hypertension, dyslipidemia, obesity, and treatment with statins and renin-angiotensin-aldosterone system [RAAS] blockade). Adequate blood pressure control was considered when the office blood pressure was < 140/90 mmHg [10]. Proper cholesterol control was considered when LDL-cholesterol was < 155 mg/dL in non-CKD patients or < 100 mg/dL in CKD patients [11].

### Statistical analysis

Statistical analyses were performed with SPSS version 20.0 software (Chicago, Ill, USA). Mean value ± standard deviation is used for quantitative data, while absolute and relative frequencies are used for qualitative variables. The Kolmogorov-Smirnoff test was used to confirm the normal distribution. The Χ^2^ test was used to compare categorical data, Student’s t-test for continuous data between two groups and ANOVA for multinomial comparisons. Cox regression analysis was used for temporal events. A significance level of 0.05 was accepted.

## Results

### ADPKD patients compared to CKDoe patients

The differences between patients with ADPKD and those with CKDoe and controls are summarized in Table 1 and supplemental Table 1 (S1). In the ADPKD group, the distribution by age and gender was similar to the controls, while patients with CKDoe, who were older and with a higher prevalence of males. In terms of comorbidities, patients with CKDoe showed more peripheral vascular disease, presence of carotid atheromatous plaque and obesity. Several differences in serum blood and urinary parameters were detected. The patients with ADPKD included in the study had worse renal function (worse mean estimated by glomerular filtration rate [eGFR], more in patients with CKD stage 4) and consequence lower hemoglobin levels, higher parathormone levels but lower proteinuria compared to CKDoe. It should be note that in the evaluation of asymptomatic CVD, patients with ADPKD had lower IMT values than the other groups, however, there was an intermediate rate of pathological ABI and atheromatous plaque.

**Table 1:** Comparison between controls, ADPKD patients and patients with CKD from other etiologies. The first “p” value corresponds to the comparison between the three groups and the second value corresponds to the comparison between ADPKD and patients with CKD from other etiologies (CKD stages 3, 4 and 5 not on dialysis)

	Controls *n* = 498 (28.4%)	ADPKD *n* = 132 (7.5%)	CKDoe *n* = 1121 (64.1%)	P Comparison three groups	P ADPKD vs CKDoe
**Age (years)**	53.69 ± 1163	53.60 ± 11.09	59.56 ± 12.22	< 0.001	< 0.001
**Male gender**	262 (52.5%)	67 (50.8%)	700 (62.1%)	< 0.001	0.012
**BMI (Kg/m**^**2**^**)**	27.82 ± 4.40	27.55 ± 4.65	28.56 ± 4.94	0.003	0.02
**Obesity (BMI > 30 Kg/m**^**2**^**)**	131 (26.3%)	35 (26.5%)	251 (34.9%)	0.010	0.03
**Active smoking**	100 (20.0%)	34 (25.7%)	212 (18.7%)	NS	NS
**Hypertension**	163 (32.7%)	123 (93.2%)	1017 (90.2%)	< 0.001	NS
**Dyslipidemia**	157 (31.5%)	82 (62.1%)	762 (67.6%)	< 0.001	NS
**CKD stage 3**	0 (0.0%)	57 (43.2%)	635 (56.3%)	< 0.001	0.004
**CKD stages 4–5**	0 (0.0%)	75 (56.8%)	493 (43.7%)	< 0.001	0.004
**Creatinine (mg/dL)**	0.84 ± 0.15	3.00 ± 1.77	2.37 ± 1.18	< 0.001	< 0.001
**eGFR (mL/min/1.73m**^**2**^**)**	91.82 ± 16.94	27.50 ± 14.09	33.46 ± 14.05	< 0.001	< 0.001
**Hemoglobin (g/dL)**	14.54 ± 1.41	12.82 ± 1.49	13.3 ± 1.73	< 0.001	0.024
**Albumin (g/dL)**	4.36 ± 0.30	4.08 ± 0.43	4.09 ± 0.45	< 0.001	NS
**Uric acid (mg/dL)**	5.05 ± 1.45	7.00 ± 1.30	6.86 ± 1.57	< 0.001	NS
**Phosphate (mg/dL)**	3.46 ± 0.52	4.02 ± 0.77	3.64 ± 0.76	< 0.001	NS
**iPTH (pg/mL)**	53.54 ± 15.34	172.26 ± 141.96	128.37 ± 110.75	< 0.001	< 0.001
**25-hydroxi-vitamin D (ng/mL)**	20.58 ± 8.39	18.13 ± 8.21	16.75 ± 7.47	< 0.001	NS
**FGF23 (pg/mL)**	53.97 ± 62.88	96.77 ± 78.44	97.71 ± 84.06	< 0.001	NS
**Potassium (mEq/L)**	4.47 ± 0.40	4.71 ± 0.51	4.75 ± 0.53	< 0.001	NS
**Proteinuria (g/day)**	0.07 ± 0.12	0.32 ± 0.38	0,98 ± 1.4	< 0.001	0.04
**Urine albumin to creatinine ratio (mg/g)**	34 ± 46	214 ± 507	427 ± 894	< 0.001	0.002
**SBP (mmHg)**	132.45 ± 17.44	140.83 ± 19.56	142.99 ± 20.73	< 0.001	NS
**DBP (mmHg)**	79.84 ± 9.66	85.14 ± 10.34	82.49 ± 10.86	< 0.001	0.006
**IMT (mm)**	0.70 ± 0.12	0.66 ± 0.11	0.72 ± 0.14	< 0.001	< 0.001
**Pathologic ABI (< 0.9 or > 1.4)**	62 (12.4%)	19 (14.4%)	244 (21.8)	< 0.001	0.027
**Ischemic ABI (< 0.9)**	56 (11.4%)	14 (11.0%)	184 (17.4%)	0.004	0.041
**Plaque presence**	248 (49.7%)	71 (53.8%)	752 (66.7%)	< 0.001	0.003
**Cardiovascular event**	11 (2.2%)	1 (0.8%)	73 (6.5%)	< 0.001	0.008
**Cardiovascular death**	0 (0.0%)	0 (0.0%)	13 (1.2%)	0.026	NS
**Non-cardiovascular death**	3 (0.6%)	3 (2.3%)	38 (3.4%)	0.004	NS

*Abbreviations*: *ABI* ankle brachial index, *ADPKD* autosomal dominant polycystic kidney disease, *BMI* body mass index, *CKD* chronic kidney disease, *CKDoe* CKD of etiologies other than ADPKD, *DBP* diastolic blood pressure, *eGFR* estimated glomerular filtration rate with the CKD-EPI formula, *FGF-23* fibroblast growth factor 23, *IMT* intima-media thickness, *iPTH* intact parathyroid hormone, *NS* not statistically significant, *SBP* systolic blood pressure

When the control of classic cardiovascular risk factors was evaluated, patients with ADPKD had the worst achievement of blood pressure targets (Table 1 and Supplemental Figure 1). The rates of overweight and obesity were like non-CKD patients. It should be note that obesity was present in 34.9% of patients with CKDoe, in 26.5% of patients with ADPKD and also in 26.3% of controls. Finally, it is also particularly noteworthy the low percentage of statins treatment in patients with CKD (Supplemental Table 2 (S2)). Only slightly more than half of the patients received statins, reaching LDL-c levels < 100 in only 50 and 39.8% of them (ADPKD and CKDoe respectively). If we consider an LDL-c target < 70 mg/dL, the percentage of patients who achieve this target is only 11 and 9.7% respectively.

**Fig. 3:**
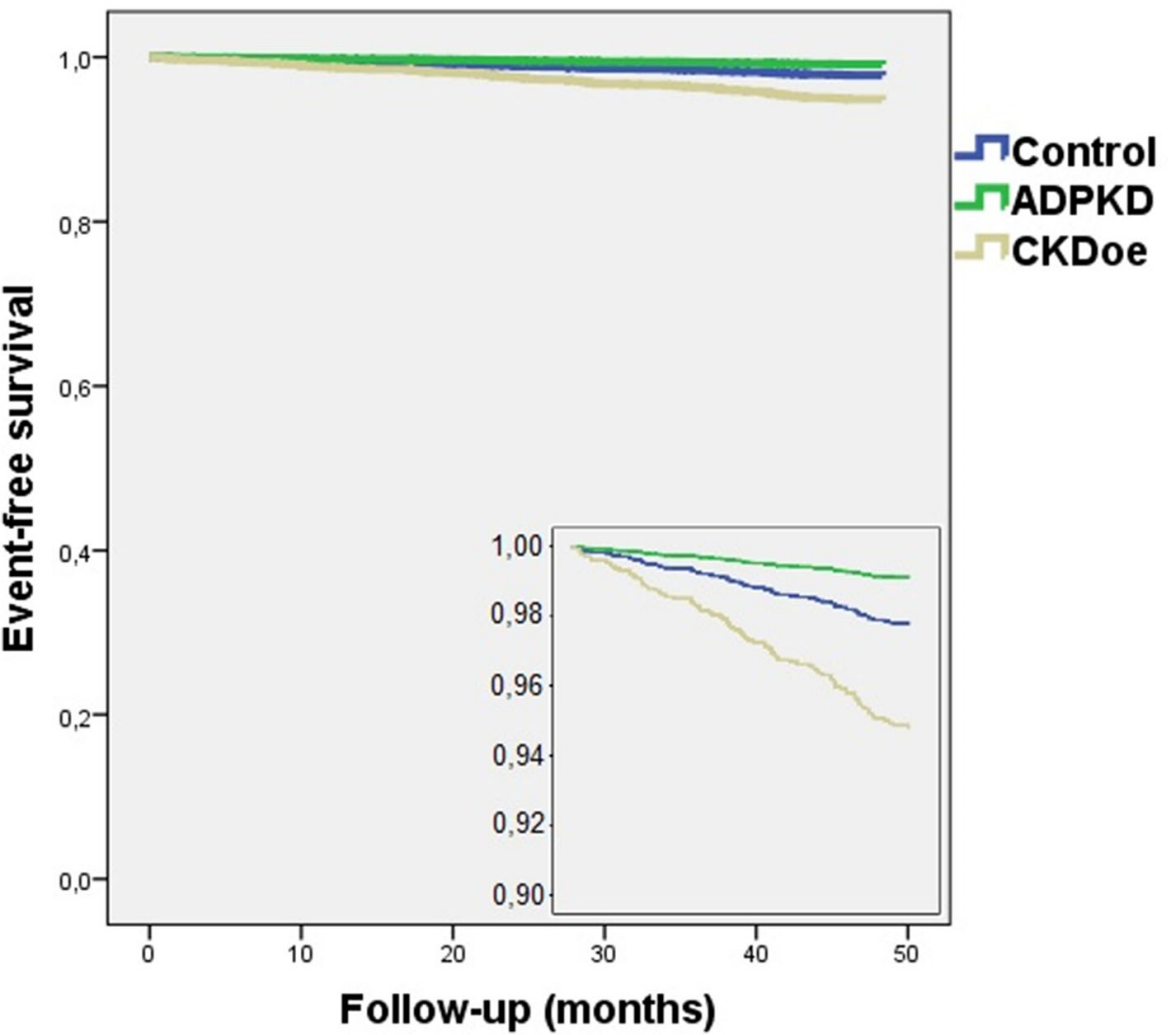
Adjusted Cox regression model of cardiovascular event free time: controls vs. ADPKD vs. CKD of other etiologies. Kaplan Meier analysis: log rank test: *p* < 0.001. Abbreviations: ADPKD, autosomal dominant polycystic kidney disease; CKD, chronic kidney disease. Cox regression model adjusted to age, sex, obesity and tobacco use

In general, the number of CVE during the 4 year of follow-up was very low (Tables 1 and 2). In a Cox regression model adjusted for age, sex, obesity, and tobacco use, ADPDK had the lowest incidence of CVE in the three groups (HR 0.422, 95%CI 0.221–0.808, *p* = 0.009) (Fig. 3).

**Table 2:** Comparison between controls and ADPKD patients with stage 3 CKD

	Controls *n* = 498 (89.7%)	ADPKD stage 3 CKD *n* = 57 (10.3%)	p
**Age (years)**	53.69 ± 11.63	54.54 ± 11.73	NS
**Male gender**	262 (52.5%)	30 (52.6%)	NS
**Hypertension**	163 (32.7%)	52 (91.2%)	< 0.001
**Dyslipidemia**	157 (31.5%)	37 (64.9%)	< 0.001
**Creatinine (mg/dL)**	0.84 ± 0.15	1.69 ± 0.35	< 0.001
**eGFR (mL/min/1.73m**^**2**^**)**	91.82 ± 16.94	41.13 ± 8.52	< 0.001
**Macroalbuminuria**	0 (0.0%)	5 (13.2%)	< 0.001
**Total cholesterol (mg/dL)**	204.41 ± 35.63	187.42 ± 40.68	0.001
**HDL-cholesterol (mg/dL)**	54.36 ± 15.38	49.05 ± 12.37	0.020
**LDL-cholesterol (mg/dL)**	128.73 ± 32.74	111.12 ± 31.93	< 0.001
**Triglycerides (mg/dL)**	112.32 ± 66.08	132.82 ± 64.99	0.030
**Hemoglobin (g/dL)**	14.54 ± 1.41	13.56 ± 1.35	< 0.001
**Albumin (g/dL)**	4.36 ± 0.30	4.23 ± 0.32	0.008
**Uric acid (mg/dL)**	5.05 ± 1.45	6.80 ± 1.28	< 0.001
**iPTH (pg/mL)**	53.54 ± 15.34	96.79 ± 46.94	< 0.001
**Potassium (mEq/L)**	4.47 ± 0.40	4.61 ± 0.45	0.017
**DBP (mmHg)**	79.84 ± 9.66	83.53 ± 9.35	0.006
**Overweight (BMI 25.1–30 Kg/m**^**2**^**)**	236 (47.3%)	23 (46.9%)	< 0.001
**Target LDL-cholesterol**	349 (80.4%)	23 (46.9%)	< 0.001
**Cardiovascular event**	11 (2.2%)	0 (0.0%)	NS
**Cardiovascular death**	0 (0.0%)	0 (0.0%)	–
**Non cardiovascular death**	3 (0.6%)	1 (1.8%)	NS

*Abbreviations*: *ABI* ankle brachial index, *ADPKD* autosomal dominant polycystic kidney disease, *BMI* body mass index, *CKD* chronic kidney disease, *DBP* diastolic blood pressure, *eGFR* estimated glomerular filtration rate with the CKD-EPI formula, *FGF-23* fibroblast growth factor 23, *HDL* high density lipoprotein, *IMT* intima-media thickness, *iPTH* intact parathyroid hormone, *LDL* low density lipoprotein, *NS* not statistically significant, *SBP* systolic blood pressure

### Stage 3 ADPKD patients compared to non-CKD subjects (control group)

A second analysis was performed, comparing only ADPKD with stage 3 CKD to non-CKD subjects, in order to better observe the specific effect of ADPKD instead of the effect of severe loss of eGFR (Table 2 and Supplemental Table 3). In this comparison, there were no significant differences in age, gender, overweight, and obesity; however, patients with ADPKD had higher rates of hypertension and dyslipidemia. As expected, due to their mild CKD, patients with ADPKD had lower levels of hemoglobin and serum albumin, and higher levels of potassium, parathormone, and uric acid. Interestingly, no differences were found in phosphate or calcium levels.

There was a lower lipid control rate among patients with ADPKD, despite a higher number of patients in statins treatment (Supplemental Table 4). Interestingly, and despite their CKD, this was not associated to higher percentages of asymptomatic CVD: no difference was found on IMT, the presence of plaque or pathologic rates of ABI. The low number of CVE during the follow-up (11 in the control group and none in the ADPKD group) did not allow for comparison.

## Discussion

To the best of our knowledge, this is the first study to assess the adequacy of the control of traditional cardiovascular risk factors in ADPKD, and their impact on asymptomatic CVD. In this large national prospective cohort, patients with ADPKD have an intermediate risk factors control rate, with a variable impact on subclinical atheromatosis but with a lower event rate compared to patients with CKDoe. Patients with ADPKD in CKD stage 3 have a similar blood pressure control, while they have a larger prevalence of hypertension and worse lipid control than patients without CKD, but a similar rate of asymptomatic CVD risk factors. It is to be noted that the classic cardiovascular risk factors are not optimally controlled.

Patients with ADPKD tend to be younger and healthier than patients with CKD who are affected from other etiologies. Although cardiovascular morbidity is lower compared to others causes of end-stage kidney disease (ESKD), patients with ADPKD continued to have higher cardiovascular morbidity and mortality [12]. A direct link between ADPKD and metabolic abnormalities leading to CVD has been proposed [13]. In addition, total kidney volume has been shown to be a predictor of CVE [14]. Hypertension occurs early in the course of the disease, among other causes due to local hyperactivation of the RAAS (Fig. 1) [15].

When a CVE occurs, it is expected to be more severe than in the general population. A study that analyzed episodes of acute myocardial infarction showed that patients with ADPKD had a higher rate of ST-segment elevation, multi-vessel involvement, and secondary sudden death [16]. However, most studies have focused on the evaluation and progression of CKD [17, 18]. Strict control blood pressure (BP) has been shown not to slow the progression of CKD, but to improve the damage in target organs [12]. However, The HALT PKD trial carried out in an early population with ADPKD showed that intensive control of systolic BP to 95–110 mmHg was associated with a 14% slower rate of kidney volume growth compared to standard control [19]. Of interest, other sub analysis of this trial found that sodium restriction is beneficial in the management of ADPKD [20].

In our study, patients with ADPKD CKD stage 3 showed a cardiovascular profile similar to controls, with similar results in ABI and even a better subclinical atheromatosis profile measured by ITM in both groups of the study with an identical mean age. This may be related to early and regular monitoring in patients with ADPKD. Being a hereditary condition, early diagnosis is frequent, leading to an early control of cardiovascular risk and the intensification of treatments for cardiovascular prevention. While this is not usually the case in the general population, as has been shown in the control group.

Data on cardiovascular risk and evaluation and prevention of CVD in patients with ADPKD is scarce. IMT represent an early marker of vascular dysfunction [21]. Several studies have shown higher IMT values in ADPKD than in healthy controls, even before the development of hypertension [22–26]. In our cohort, patients with ADPKD had the lowest IMT, while patients with ADPKD CKD stage 3 presented a similar IMT to controls. Previous studies have shown very similar values of IMT, although they were performed in patients with ADPKD with normal renal function [23]. This difference can be explained by the fact that, for the present study, the controls were not completely healthy subjects, but patients recruited from primary care with different comorbidities.

The presence of plaque in the carotid or femoral arteries may be considered established CVD, a late-stage or asymptomatic CVD [27]. In our study, patients with ADPKD had an intermediate rate of plaque presence between controls and subjects with CKDoe, while stage 3 CKD ADPKD was not associated with higher rates of atheromatous plaques than controls. As far as we know, no other study has reported similar findings. Asymptomatic peripheral vascular disease is relatively common in CKD [28]. ABI is a proved marker of CVD, and both low and high values have shown to predict CVE in patients with CKD [29]. Pathologic rates of ABI values were lower in patients with ADPKD than in CKDoe, and similar to controls without CKD. A previous study showed a normal ABI value in patients with ADPKD with an eGFR > 60 mL/min [30].

In this cohort, BP and weight control were not lower than controls or patients with CKDoe, as long as BP control is understood as for general CKD patients, not as for the intense control recommended for ADPKD patients [17]. Despite more frequent use of statins, LDL control was lower than controls and similar to other patients with CKD. One possible explanation could be the use of insufficient statin doses. In general terms, the adequacy of the control of cardiovascular risk factors in patients with ADPKD observed in our study may be responsible for the low rates of asymptomatic CVD. Lipid control also appears to affect the progression of CKD in ADPKD; however, the actual pleiotropic effect of statins remains unclear [17, 31]. Other studies have shown worse lipid control and higher uric acid levels and inflammatory markers, also not associated with worse IMT or ABI [30]. These results could be influenced not only by serum lipid levels, but also by deeper lipid disturbances, such as lipid particle number, size and density. These alterations have been related to CVD in CKD of other etiologies [32], and recently preliminary data have been published in this regard in patients with ADPKD [33].

The small differences between patients with ADPKD at stage 3 of CKD and controls support the hypothesis that the main cause for CVD in the ADPKD population is CKD rather than the disease itself. While recognizing that the control population is not completely healthy, its cardiovascular status is similar to that of patients with CKD stage 3 ADPKD, suggesting that a good control of the risk factors for CV in ADPKD should allow ESKD to be achieved in a good cardiovascular health.

Therefore, there is scope to improve cardiovascular risk control in ADPKD, although most indications have a low level of evidence based on expert opinions [34, 35]. Recent CVD guidelines [36] have established the CKD condition as high or very high risk with the BP target below 140/90 with a tendency to a systolic BP of 130 in CKD if tolerated [36]. In our study, only half of the patients achieved the less strict BP target. Recently the ESC/EAS Guidelines for the management of dyslipidemias have also considered CKD patients as high or very high risk with LDL cholesterol objectives of 70 mg/dL and 55 mg/dL respectively [37], being these levels far from the data shown in our study. The high prevalence of overweight and obesity in patients with CKD, including patients with ADPKD, coupled with the lack of achievement of BP and lipid targets means that much remains to be done. These data show the need to increase awareness about intensifying the control of classic cardiovascular risk factors in patients with ADPKD. It does not make much sense to use expensive disease modifying drugs without using effective adjuvant therapies that prevent CVE.

A deeper view of CVD and atheromatous disease is needed in the near future. In this study, patients with ADPKD-derived CKD showed a similar or even lower long-term cardiovascular risk than those with or without CKD. Given the condition of hereditary disease, in most cases patients with ADPKD have benefited from routine vascular evaluation tests because they have begun their follow-up from the early stages. This have demonstrated a prognostic effect, as we have shown in our study. However, we are still far from achieving the optimal objectives for the control of cardiovascular risk factors.

Finally, a major effort must be made to look beyond the prevention of the progression of CKD, towards a more intense management of cardiovascular risk, which should lead to a reduction in CVE, especially if it could be implemented early in the course of the disease.

Our study has several limitations. The main one is the existence of an intentional selection bias: since patients with previous CVE were excluded from the study, included patients had a starting lower cardiovascular profile. Besides, non-CKD patients are not matched healthy controls. However, the control group had a mean age like those in the ADPKD group. Finally, there were many dropouts through the follow-up, mainly due to kidney transplantation. Another limitation is related to relatively low patients with ADPKD included. The study also has important strengths, being the main its large sample, and its multicenter and prospective nature.

In conclusion, patients with ADPKD show intermediate control rates of cardiovascular comorbidities, when compared to non-CKD subjects and other etiologies of CKD. Better control of cardiovascular risk factors appears to be associated with a lower burden of CVD, which may lead in the long term to a better prognosis. However, we are still far from achieving the optimal goals for the control of cardiovascular risk factors in patients with ADPKD. Further investigation is needed to deepen our knowledge about the course of CVD in ADPKD and to determine the usefulness of specific therapeutic measures to improve cardiovascular prognosis in ADPKD.

## Supplementary Information

**Additional file 1.**

## Data Availability

All data generated or analyzed during this study are included in this published article.
