# Supplementary material for: Cardiovascular risk factors and the impact on prognosis in patients with chronic kidney disease secondary to autosomal dominant polycystic kidney disease
Source: BMC Nephrol. 2021 Mar 25;22:110. doi: 10.1186/s12882-021-02313-1 (PMC7995703; doi:10.1186/s12882-021-02313-1)
Supplement: Supplementary file 1 — Additional file 1. [file 12882_2021_2313_MOESM1_ESM.docx]

**CARDIOVASCULAR RISK FACTORS AND THE IMPACT ON PROGNOSIS IN PATIENTS WITH CHRONIC KIDNEY DISEASE SECONDARY TO AUTOSOMAL DOMINANT POLYCYSTIC KIDNEY DISEASE**

José Luis Gorriz^1^, David Arroyo^2^, Luis D’Marco^1^, Roser Torra^3^, Patricia Tomás^1^, María Jesús Puchades^1^, Nayara Panizo^1^, Jonay Pantoja^4^, Marco Montomoli^1^, José Luis Llisterri^5^, Vicente Pallares-Carratalá^6^, José Manuel Valdivielso^7^.

**Supplemental Table 1.** Comparison between controls, ADPKD patients and patients with CKD from other etiologies. The first "p" value corresponds to the comparison between the three groups and the second value corresponds to the comparison between ADPKD and patients with CKD from other etiologies (CKD stages 3, 4 and 5 not on dialysis).

|  | **Controls**  **n=498 (28.4%)** | **ADPKD**  **n=132 (7.5%)** | **CKDoe**  **n=1121 (64.1%)** | **P**  Comparison three groups | **P**  ADPKD vs CKDoe |
| --- | --- | --- | --- | --- | --- |
| **Weight (Kg)** | 76.00±14.65 | 76.85±14.86 | 76.88±14.47 | NS | NS |
| **Waist circumference (cm)** | 95.90±10.86 | 96.18±11.11 | 98.09±12.14 | 0.001 | NS |
| **Familial history of CVD** | 58 (11.6) | 14 (10.6) | 94 (8.4) | NS | NS |
| **Macroalbuminuria** | 0 (0.0%) | 18 (21.7%) | 257 (33.8%) | <0.001 | <0.001 |
| **Total cholesterol (mg/dL)** | 204.41±35.63 | 181.80±37.37 | 187.39±37.19 | <0.001 | NS |
| **HDL-cholesterol (mg/dL)** | 54.36±15.38 | 49.70±13.52 | 51.69±15.32 | 0.001 | NS |
| **LDL-cholesterol (mg/dL)** | 128.73±32.74 | 106.57±32.26 | 109.73±32.39 | <0.001 | NS |
| **Triglycerides (mg/dL)** | 112.32±66.09 | 127.98±67.17 | 138.32±72.96 | <0.001 | NS |
| **Apo A1 (g/L)** | 1.24±0.11 | 1.42±0.35 | 1.58±0.39 | NS | NS |
| **Apo B (g/L)** | 0.80±0.12 | 0.81±0.18 | 0.88±0.25 | NS | NS |
| **Glucose (mg/dL)** | 96.20±12.92 | 93.55±11.67 | 96.61±13.78 | 0.045 | NS |
| **Total calcium (mg/dL)** | 9.41±0.43 | 9.38±0.53 | 9.40±0.54 | NS | NS |
| **Insulin (μU/mL)** | 5.99±4.86 | 9.84±6.51 | 10.51±9.56 | <0.001 | NS |
| **SBP >140 mmHg** | 152 (30.5%) | 55 (41.7%) | 570 (50.5%) | <0.001 | NS |
| **DBP >90 mmHg** | 70 (14.0%) | 34 (25.8%) | 231 (20.5%) | 0.001 | NS |
| **SBP >140 + DBP <90 mmHg** | 102 (20.4%) | 33 (25.0%) | 409 (36.3%) | <0.001 | 0.036 |
| **SBP >140 + DBP >90 mmHg** | 60 (12.0%) | 28 (21.2%) | 196 (17.4%) | <0.001 | 0.04 |
| **Overweight (BMI 25.1-30 Kg/m^2^)** | 236 (47.3%) | 58 (43.9%) | 483 (42.8%) | NS | NS |
| **Target LDL-cholesterol** | 349 (80.4%) | 59 (50.0%) | 390 (39.8%) | <0.001 | 0.022 |

**Abbreviations:** ADPKD, autosomal dominant polycystic kidney disease; CKD, chronic kidney disease; CKDoe, CKD of etiologies other than ADPKD; DBP, diastolic blood pressure; HDL, high density lipoprotein; LDL, low density lipoprotein; NS, not statistically significant; SBP, systolic blood pressure.

**Supplemental table 2.** Comparison of treatment between controls, ADPKD patients and patients with CKD from other etiologies. The first "p" value corresponds to the comparison between the three groups and the second value corresponds to the comparison between ADPKD and patients with CKD from other etiologies (CKD stages 3, 4 and 5 not on dialysis).

|  | **Controls**  **n=498 (28.4%)** | **ADPKD**  **n=132 (7.5%)** | **CKDoe**  **n=1121 (64.1%)** | **P**  Comparison three groups | **P**  ADPKD vs CKDoe |
| --- | --- | --- | --- | --- | --- |
| **treatments** | 158 (31.7%) | 125 (94.7%) | 1037 (91.9%) | <0.001 | NS |
| **RAAS blockade** | 122 (24.4%) | 103 (78.1%) | 869 (76.2%) | <0.001 | NS |
| **ACE inhibitors** | 41 (8.2%) | 41 (31.1%) | 393 (34.8%) | <0.001 | NS |
| **Angiotensin II receptor blockers** | 83 (16.6%) | 72 (54.5%) | 597 (52.9%) | <0.001 | NS |
| **Calcium channel blockers** | 43 (8.6%) | 46 (34.8%) | 381 (33.8%) | <0.001 | NS |
| **Diuretics** | 34 (6.8%) | 50 (37.9%) | 518 (45.9%) | <0.001 | NS |
| **Alfa blockers** | 14 (2.8%) | 21 (15.9%) | 195 (17.3%) | <0.001 | NS |
| **Beta blockers** | 20 (4.0%) | 17 (12.9%) | 167 (14.8%) | <0.001 | NS |
| **Fibrates** | 11 (2.2%) | 3 (2.3%) | 67 (5.9%) | 0.002 | NS |
| **Statin treatment** | 103 (20.6%) | 76 (57.6%) | 677 (60.0%) | <0.001 | NS |
| **Ezetimibe** | 6 (1.2%) | 3 (2.3%) | 52 (4.6%) | 0.002 | NS |
| **Anti-aggregation** | 28 (5.6%) | 4 (3.0%) | 157 (13.9%) | <0.001 | <0.001 |
| **Anticoagulation** | 2 (0.4%) | 1 (0.8%) | 41 (3.6%) | <0.001 | NS |
| **Erythropoiesis stimulating agents** | 0 (0%) | 100 (44.4%) | 562 (35.1%) | <0.001 | NS |

**Abbreviations:** ACE, angiotensin-converting enzyme; ADPKD, autosomal dominant polycystic kidney disease; CKD, chronic kidney disease; CKDoe, CKD of etiologies other than ADPKD; NS, not statistically significant; RAAS, renin-angiotensin-aldosterone system; ESA: erythropoiesis stimulating agents.

**Supplemental Table 3.** Comparison between controls and ADPKD patients with stage 3 CKD.

|  | **Controls**  **n=498 (89.7%)** | **ADPKD stage 3 CKD**  **n=57 (10.3%)** | **p** |
| --- | --- | --- | --- |
| **Weight (Kg)** | 76.00±14.65 | 78.21±14.90 | NS |
| **BMI (Kg/m^2^)** | 27.82±4.40 | 28.27±5.14 | NS |
| **Waist circumference (cm)** | 95.90±10.86 | 97.14±10.73 | NS |
| **Active smoking** | 100 (20.0%) | 16 (28.0%) | NS |
| **Atrial fibrillation** | 1 (0.2%) | 0 (0.0%) | NS |
| **Apo A1 (g/L)** | 1.24±0.11 | 1.68±0.28 | NS |
| **Apo B (g/L)** | 0.80±0.12 | 0.86±0.12 | NS |
| **Glucose (mg/dL)** | 96.20±12.92 | 93.78±9.89 | NS |
| **Insuline (μU/mL)** | 5.99±4.86 | 10.17±4.54 | NS |
| **Total calcium (mg/dL)** | 9.41±0.43 | 9.41±0.49 | NS |
| **Phosphate (mg/dL)** | 3.46±0.52 | 3.59±0.49 | NS |
| **25-hydroxi-vitamin D (ng/mL)** | 20.58±8.39 | 18.39±10.04 | NS |
| **SBP (mmHg)** | 132.45±17.44 | 137.00±20.20 | NS |
| **SBP >140 mmHg** | 152 (30.5%) | 20 (35.1%) | NS |
| **DBP >90 mmHg** | 70 (14%) | 12 (21.1%) | NS |
| **SBP >140 + DBP <90 mmHg** | 102 (20.4%) | 12 (21.1%) | NS |
| **SBP >140 + DBP >90 mmHg** | 60 (12.0%) | 10 (17.5%) | NS |
| **Obesity (BMI >30 Kg/m^2^)** | 131 (26.3%) | 19 (33.3%) | NS |
| **IMT (mm)** | 0.70±0.14 | 0.69±0.13 | NS |
| **Pathologic ABI (<0.9 or >1.4)** | 62 (12.4%) | 8 (14.0%) | NS |
| **Ischemic ABI (<0.9)** | 56 (11.4%) | 7 (12.5%) | NS |
| **Plaque presence** | 248 (49.7%) | 31 (54.4%) | NS |
| **Beta blockers** | 20 (4.0%) | 4 (7.0%) | NS |
| **Fibrates** | 11 (2.2%) | 1 (1.8%) | NS |
| **Ezetimibe** | 6 (1.2%) | 1 (1.8%) | NS |
| **Antiaggregating** | 28 (5.6%) | 2 (3.5%) | NS |
| **Anticoagulation** | 2 (0.4%) | 0 (0.0%) | NS |

**Abbreviations:** ADPKD, autosomal dominant polycystic kidney disease; CKD, chronic kidney disease; CKDoe, CKD of etiologies other than ADPKD; DBP, diastolic blood pressure; HDL, high density lipoprotein; LDL, low density lipoprotein; NS, not statistically significant; SBP, systolic blood pressure.

**Supplemental Table 4.** Comparison the treatment between controls and ADPKD patients with stage 3 CKD.

| **Treatment** | **Controls**  **n=498 (89.7%)** | **ADPKD stage 3 CKD**  **n=57 (10.3%)** | **p** |
| --- | --- | --- | --- |
| **Statin treatment** | 103 (20.6%) | 34 (59.6%) | <0.001 |
| **Antihypertensive treatment** | 158 (31.7%) | 53 (93.0%) | <0.001 |
| **RAAS blockade** | 122 (24.4%) | 48 (84.2%) | <0.001 |
| **ACE inhibitors** | 41 (8.2%) | 20 (35.1%) | <0.001 |
| **Angiotensin II receptor blockers** | 83 (16.6%) | 32 (56.1%) | <0.001 |
| **Calcium channel blockers** | 43 (8.6%) | 15 (26.3%) | <0.001 |
| **Diuretics** | 34 (6.8%) | 21 (36.8%) | <0.001 |
| **Alfa blockers** | 14 (2.8%) | 7 (12.3%) | <0.001 |

**Abbreviations:** ACE, angiotensin-converting enzyme; ADPKD, autosomal dominant polycystic kidney disease; CKD, chronic kidney disease; CKDoe, CKD of etiologies other than ADPKD; NS, not statistically significant; RAAS, renin-angiotensin-aldosterone system; ESA: erythropoiesis stimulating agents.

**Supplemental Figure 1.** Control rates of cardiovascular risk factors: controls vs. ADPKD vs. CKD of other etiologies.


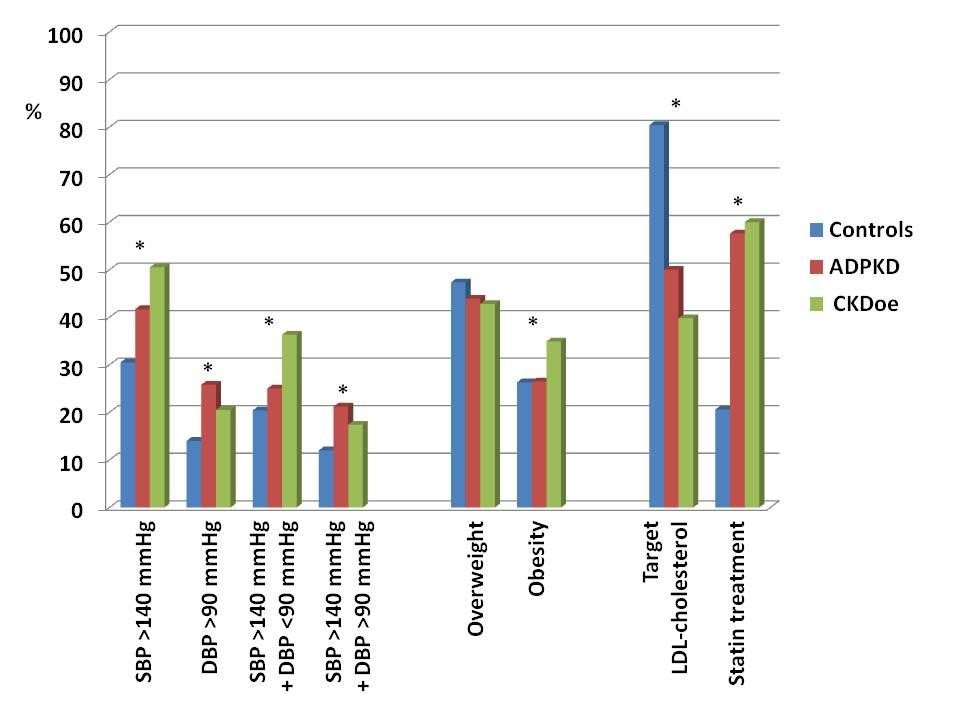


**Abbreviations:** ADPKD, autosomal dominant polycystic kidney disease; CKD, chronic kidney disease; CKDoe, CKD of etiologies other than ADPKD. * Statistically significant differences.
